# 

**Published:** 1817-03

**Authors:** 


### CATALOGUE OF MEDICAL BOOKS.

Memoirs of the Life and Doctrine of the late John Hunter, Esq. Founder of the Hunterian Museum in the College of Surgeons. By Joseph Adams, M.D. Author of Observations on Morbid Poisons. 8vo.

A General System of Toxicology, or a Treatise on Poisons drawn from the Mineral, Vegetable, and Animal Kingdoms, considered as to their relations with Physiology, Pathology, and Medical Jurisprudence. By M. P. Orfila, M.D. &c. Translated from the French. Vol. 2, part 1; 8vo.

Elements of Chemistry. By John Murray, M.D. &c. The 4th edit. in 2 vols. 8vo.

The Hospital Pupil; or Observations addressed to the Parents of Youths intended for the Profession of Medicine or Surgery, on their previous Education, pecuniary Resources, and on the order of their Professional Studies; with Hints to the young Pupil, on the prosecution of Hospital Studies, on entering into Practice, and on Medical Jurisprudence. By James Parkinson, Member of the Royal College of Surgeons. 2d edit. 12mo.

A Cursory Inquiry into some of the principal Causes of Mortality among Children, with a view to assist in ameliorating the State of the rising Generation, in Health, Morals, and Happiness: to which is added, an Account of the Universal Dispensary for Sick Indigent Children. By John Bunnell Davis, M.D. &c. 8vo.

The Anatomy of the Human Bones and Nerves, with a Description of the Human Lacteal Sac and Duct. By Alexander Monro, M.D. &c. Carefully Revised, with additional Notes and Illustrations; by Jeremiah Kirby, M.D. &c. 2d edit. 12mo.

---

### *NOTICES TO CORRESPONDENTS.*

*We wish our Correspondents, in future, would favour us with the designation by which their Communications may be described. For want of this, we have erroneously given a heading to* Dr. Yeats's *paper in our last Number. That gentleman's only intention was to explain, not to accuse the Reviewer, in a highly respectable journal, of any want of candour. On this subject we have received a letter from the Doctor, very much regretting that such an improper heading should be given to his paper.*

*We thank* Æquus *for his good intention, but his paper is written in such a hurry as to render it not perfectly intelligible.*

Mr. C.'s *Letter shall be attended to. By some accident, the paper he alludes to had been mislaid.*

Mr. Spencer's *Paper shall appear in our next.*

*We presume our correspondent* Hint *cannot be ignorant that, in the year* 1779, *such a work as he proposes was published by Dr. S. F. Simmons. In* 1783, *it was re-published; and, in the Preface, we see a reference to two former editions. It was published first by Murray, in Fleet-street, and afterwards by Johnson, in St. Paul's Church Yard.*

